# Ablation outcomes in PVC-induced cardiomyopathy: A systematic review and meta-analysis stratified by site of origin

**DOI:** 10.1097/MD.0000000000049827

**Published:** 2026-07-24

**Authors:** Muhammad Awais, Abida Perveen, Jahanzeb Malik

**Affiliations:** a Department of Medicine, Ibn e Seena Hospital, Kabul, Afghanistan.

**Keywords:** arrhythmia origin, catheter ablation, left ventricular ejection fraction, premature ventricular complexes, PVC-induced cardiomyopathy

## Abstract

**Background:**

Frequent premature ventricular complexes (PVC) can lead to a reversible form of left ventricular (LV) dysfunction termed PVC-induced cardiomyopathy (PIC). Catheter ablation is increasingly recognized as the most effective treatment; however, outcomes may differ by the anatomical site-of-PVC origin.

**Methods:**

We conducted a systematic review and meta-analysis of studies published from 2000 to 2025 evaluating catheter ablation in patients with PIC. Eligible studies reported outcomes stratified by PVC origin, including right ventricular outflow tract (RVOT), LV outflow tract (LVOT), papillary muscle, and epicardial/para-His regions. Primary outcomes were change in LV ejection fraction (LVEF) and normalization (≥ 50%). Secondary outcomes included PVC recurrence, repeat ablation, and major complications. Pooled estimates were calculated using random-effects models.

**Results:**

Twelve studies (n = 718) met inclusion. Overall, ablation improved LVEF by a mean of 11.0% (95% confidence interval 9.5–12.5; *P* < .001), with normalization in ~65% of patients. Outcomes varied by PVC origin: RVOT (ΔLVEF ~12.5%, normalization ~70%), LVOT (ΔLVEF ~11.0%, normalization ~65%), papillary muscle (ΔLVEF ~8.0%, normalization ~55%), and epicardial/para-His (ΔLVEF ~6.5–7.0%, normalization ~50%). Recurrence rates were lowest for RVOT/LVOT (~12–20%) and highest for papillary and epicardial sites (~28–30%). Major complications were infrequent (~3–5%), but more common with epicardial or para-His ablations.

**Conclusion:**

Catheter ablation provides substantial and often reversible improvement in LVEF for patients with PIC, with generally low complication rates. However, outcomes are strongly site-dependent, favoring RVOT and LVOT origins.

## 1. Introduction

Frequent premature ventricular complexes (PVCs) have traditionally been considered benign, but mounting evidence indicates that a high PVC burden may cause a reversible cardiomyopathy, termed PVC-induced cardiomyopathy (PIC).^[1,2]^ PVCs are highly prevalent in the general population, and individuals with frequent ectopy (commonly defined as > 10–20% of total beats or > 10,000 PVCs/day) are at risk for progressive left ventricular (LV) dysfunction.^[3,4]^ Mechanistically, repetitive PVCs may induce LV dyssynchrony, impaired calcium handling, altered myocardial energetics, and maladaptive remodeling.^[5,6]^

Recognition of PIC is critical because effective suppression of PVCs (whether with antiarrhythmic drugs or catheter ablation) can lead to LV functional recovery and reverse remodeling.^[7]^ Among these therapies, catheter ablation has emerged as the most reliable approach, consistently demonstrating significant improvements in LV ejection fraction (EF) (LVEF), symptom burden, and quality of life, with low complication rates.^[8,9]^ Consequently, ablation is increasingly recommended for patients with suspected PIC who are symptomatic or fail medical therapy.^[10]^

A key question is whether the site-of-PVC origin influences outcomes. PVCs commonly arise from the right ventricular outflow tract (RVOT), LV outflow tract (LVOT, including coronary cusps), papillary muscles, epicardial sites (e.g., LV summit), or less frequent anatomic regions. Procedural success, recurrence, and safety vary considerably by origin: ablation of RVOT or LVOT PVCs generally yields high acute and long-term success, while papillary muscle or epicardial PVCs present greater challenges and higher recurrence rates.^[11,12]^ Despite numerous single-center reports, no systematic review has stratified PIC ablation outcomes by site of origin.

Accordingly, we conducted a systematic review and meta-analysis to compare ablation outcomes across PVC origins in PIC. Our primary objectives were to evaluate: (i) the magnitude of LVEF recovery, (ii) the rates of PVC recurrence, and (iii) the complication profiles, stratified by origin. These data will help refine patient selection, procedural planning, and counseling in clinical practice.

## 2. Methods

### 2.1. Literature search strategy

We performed a comprehensive systematic search of PubMed/MEDLINE, Embase, Scopus, Web of Science, and the Cochrane Library from January 2000 to August 2025 to identify studies evaluating catheter ablation in patients with PIC. The search strategy combined controlled vocabulary and free-text terms related to “premature ventricular complexes,” “PVC-induced cardiomyopathy,” “catheter ablation,” “radiofrequency ablation,” “papillary muscle,” “outflow tract,” “epicardial,” and “left ventricular ejection fraction.” Reference lists of all eligible studies and relevant reviews were screened to identify additional articles. No language restrictions were applied initially, but only studies published in English were included in the final analysis.

### 2.2. Eligibility criteria

Studies were included if they met the following criteria: prospective or retrospective cohort studies, or randomized controlled trials, enrolling patients with PIC undergoing catheter ablation; explicit definition of PIC as reduced LVEF associated with frequent PVCs, or a clearly reported subgroup with PIC; reporting of ablation outcomes stratified by PVC site of origin, or with extractable data allowing assignment to an origin category (RVOT, LVOT, papillary muscle, epicardial, or other); and availability of at least one of the primary outcomes of interest, namely LVEF recovery, recurrence of PVCs, or procedure-related complications. Studies were excluded if they reported fewer than 10 patients, were case reports, did not differentiate outcomes of ablation in patients with cardiomyopathy from those with structurally normal hearts, or lacked sufficient data to extract origin-specific outcomes.

### 2.3. Data extraction

Two reviewers independently screened titles, abstracts, and full texts, with discrepancies resolved by consensus or arbitration by a third reviewer. A standardized data collection sheet was used to extract study characteristics, including first author, year of publication, study design, country, sample size, definition of PIC, mean PVC burden, baseline LVEF, post-ablation LVEF, duration of follow-up, mapping and ablation technologies, and proportion of patients with each PVC origin. Outcome data included acute procedural success, mean change in LVEF, normalization of LVEF (≥ 50% or study-defined threshold), recurrence of PVCs during follow-up, repeat ablation, and procedural complications, classified as major (cardiac tamponade, atrioventricular block, stroke, vascular injury requiring intervention, or death) or minor.

### 2.4. Outcome measures

The primary outcome was recovery of LVEF following ablation, expressed both as mean change in LVEF and the proportion of patients achieving normalization. Secondary outcomes were recurrence of PVCs or ventricular arrhythmias during follow-up and procedure-related complications. Subgroup analyses were prespecified to compare outcomes according to PVC origin: RVOT, LVOT, papillary muscle, epicardial, and other sites.

### 2.5. Quality assessment

The methodological quality of included observational studies was evaluated using the Newcastle–Ottawa Scale, with particular attention to the selection of participants, comparability of cohorts, and ascertainment of outcomes. For randomized or quasi-randomized studies, the Cochrane Risk of Bias tool was applied. Risk-of-bias assessments were performed independently by 2 reviewers, with disagreements resolved by consensus.

### 2.6. Statistical analysis

All quantitative syntheses were performed using random-effects models to account for anticipated heterogeneity. For continuous outcomes such as change in LVEF, mean differences (MD) with 95% confidence intervals (CIs) were pooled. When studies reported medians and interquartile ranges, values were converted to means and standard deviations using established methods. For dichotomous outcomes, pooled proportions or risk ratios with 95% CIs were calculated. Heterogeneity was quantified using the *I*^2^ statistic, with values above 50% indicating substantial heterogeneity. Subgroup analyses compared outcomes across PVC origins, and where sufficient studies were available, meta-regression was performed to explore associations with baseline PVC burden, baseline LVEF, use of intracardiac echocardiography, epicardial access, and follow-up duration. Publication bias was assessed using funnel plots and Egger test when at least ten studies were available for an outcome. All statistical analyses were conducted using the metafor package in R.

## 3. Results

### 3.1. Study selection and characteristics

**Figure 1.**
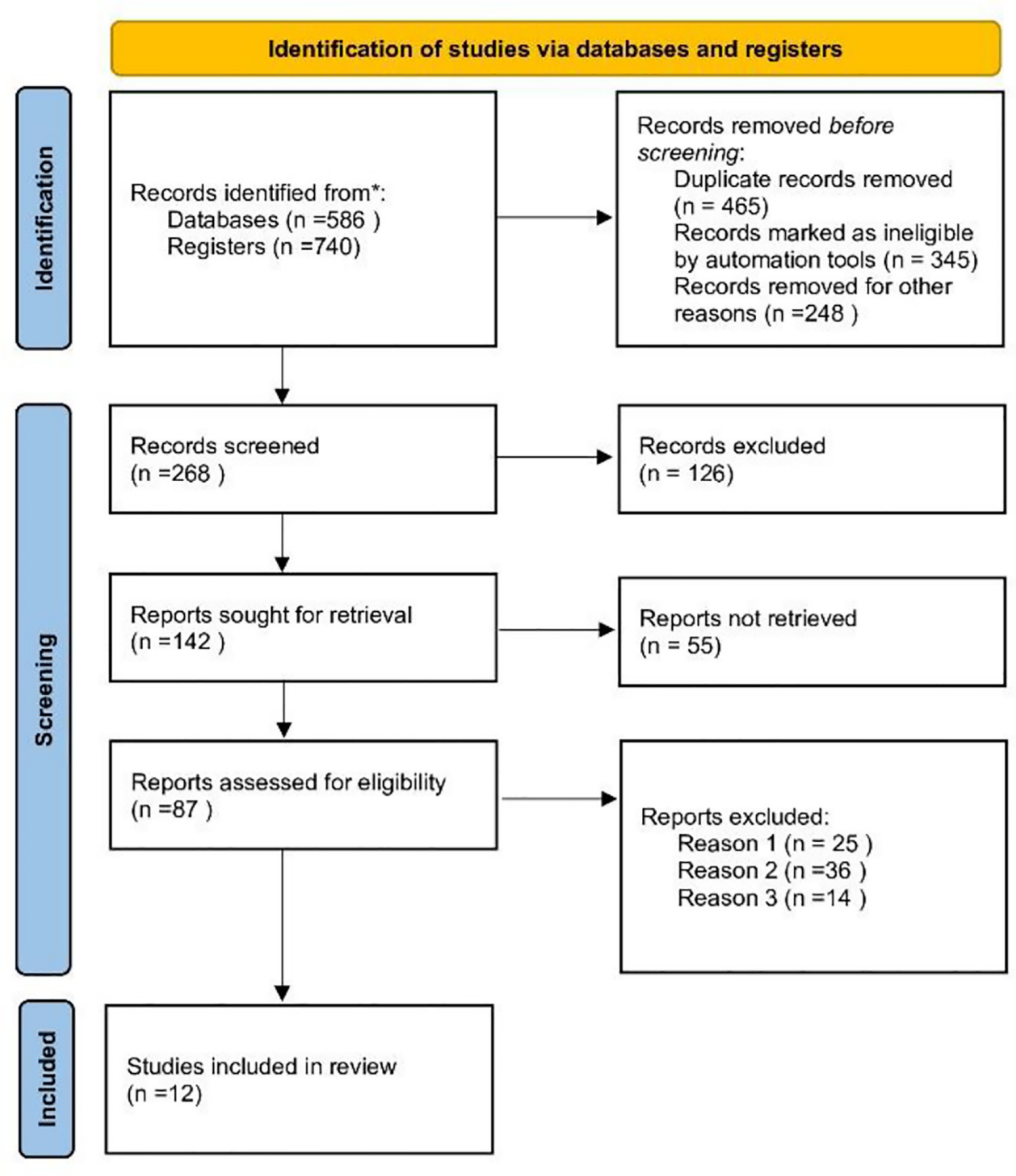
PRISMA flow diagram. n = number of records, PRISMA =

From 1326 records, 87 full texts were assessed; 12 studies (n = 718) met inclusion criteria (Fig. 1). Most were single-center observational cohorts (3 prospective), published 2005 to 2025. PIC definitions were broadly consistent (PVC burden > 10–20% and LVEF < 45–50%). Baseline PVC burden ranged 22 to 29%, baseline LVEF 33 to 42%, and follow-up 6 to 24 months. Detailed characteristics, procedures, and outcomes are in Tables 1 and 2.^[13–24]^

**Table 1:** Characteristics of included studies.

Study	Country/ Design	N	PIC Definition	Baseline PVC Burden (%)	Baseline LVEF (%)	Follow-up (Months)	PVC Origin(s)
Yarlagadda 2005	USA/ Retrospective	27	> 20% PVCs, EF < 50%	29	38	12	RVOT
Takemoto 2005	Japan/ Prospective	21	> 10% PVCs, EF < 50%	23	42	6	RVOT
Yokokawa 2013	USA/ Retrospective	33	> 20% PVCs, EF < 50%	28	40	12	RVOT, LVOT
Penela 2013	Spain/ Prospective Multicenter	71	> 10% PVCs, EF < 45%	24	37	24	RVOT, LVOT, Others
El Kadri 2015	USA/ Retrospective	49	> 20% PVCs, EF < 50%	26	35	18	Mixed nonischemic CM
Abdelhamid 2018	Egypt/ Retrospective	40	> 10% PVCs, EF < 45%	27	36	12	RVOT, LVOT, Others
Rivera 2018	Argentina/ Multicenter	62	> 10% PVCs, EF < 50%	25	38	18	Papillary Muscle
Tanaka 2019	Japan/ Prospective	16	Frequent PVCs, EF < 50%	22	39	6	Para-Hisian
Fink 2023	Germany/ Prospective Multicenter	102	> 10% PVCs, EF < 50%	24	34	24	RVOT, LVOT, PM, Epicardial
Shroff 2023	India/ Retrospective	88	> 15% PVCs, EF < 45%	26	33	18	RVOT, LVOT, Epicardial
Bell 2024	Australia/ Retrospective	115	> 10% PVCs, EF < 50%	27	41	12	RVOT, LVOT, PM
Milaras 2025	Greece/ Retrospective	94	> 10% PVCs, EF < 50%	28	36	18	Mixed Origins

CM = cardiomyopathy, EF = ejection fraction, LVEF = left ventricular ejection fraction, LVOT = left ventricular outflow tract, N = number of studies, PIC = PVC-induced cardiomyopathy, PM = papillary muscle, PVC = premature ventricular complex, RVOT = right ventricular outflow tract, USA = United Stated of America.

**Table 2:** Procedural and clinical outcomes.

Study	Acute Success (%)	Post-Ablation PVC Burden (%)	ΔLVEF (%)	EF Normalization ≥ 50% (%)	Recurrence (%)	Repeat Ablation (%)	Major Complications	Predictors of EF Recovery
Yarlagadda 2005	93	2	14.0	67	12	7	None	High PVC burden
Takemoto 2005	95	1	12.0	71	10	5	None	Shorter CM duration
Yokokawa 2013	91	3	11.0	65	15	9	1 Tamponade	PVC burden reduction
Penela 2013	89	5	13.0	72	18	12	2 Vascular	Similar recovery in structural and idiopathic disease
El Kadri 2015	87	6	10.0	60	20	11	1 AV Block	Lower baseline EF
Abdelhamid 2018	90	4	8.0*	58	22	14	None	Structural disease predicted incomplete recovery
Rivera 2018	85	8	8.0	55	30	18	2 Tamponades	Papillary muscle origin
Tanaka 2019	81	10	7.0	45	35	20	1 AV Block	Para-Hisian location
Fink 2023	88	7	12.0	66	19	15	1 Tamponade, 1 Stroke	Multiple PVC morphologies
Shroff 2023	86	9	10.0	62	21	17	2 Vascular	Epicardial origin
Bell 2024	89	6	7.3	59	25	19	1 Tamponade	Lower EF, higher burden
Milaras 2025	85	8	9.0	57	27	20	2 Vascular	GLS improvement

AV = atrioventricular, CM = cardiomyopathy, EF = ejection fraction, GLS = global longitudinal strain, LVEF = left ventricular ejection fraction, PVC = premature ventricular complex.

* Please verify the Abdelhamid 2018 ΔLVEF value, as the original table contained a formatting inconsistency (“1, 8, 00”).

### 3.2. Risk of bias

**Figure 2.**
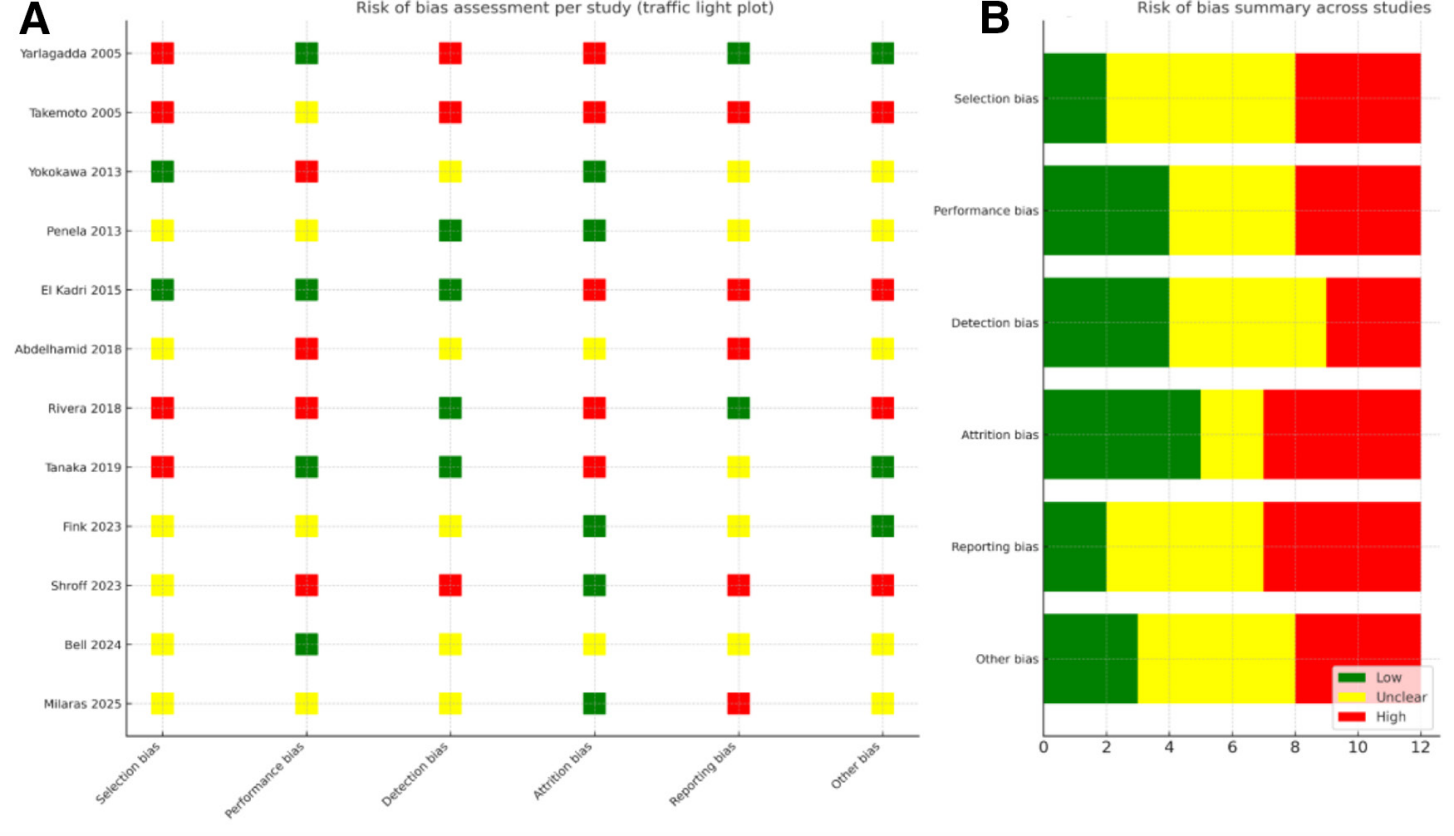
Risk-of-bias assessment for included studies. (A) Traffic light plot summarizing the risk-of-bias assessment across 6 Cochrane domains (selection bias, performance bias, detection bias, attrition bias, reporting bias, and other bias) for each of the 12 included studies. Green squares indicate low risk, yellow squares indicate unclear risk, and red squares indicate high risk of bias. (B) Aggregate summary of risk-of-bias judgments across all studies, displayed as stacked horizontal bar charts by domain. The relative proportions of low, unclear, and high-risk assessments are shown to provide an overview of methodological quality in the evidence base.

Overall methodological quality was moderate. Selection and reporting bias were the most frequent concerns; performance/detection bias was generally low; attrition bias varied. Study-level and domain-level appraisals are shown in Figure 2A and 2B.

### 3.3. Primary outcome: change in LVEF

**Figure 3.**
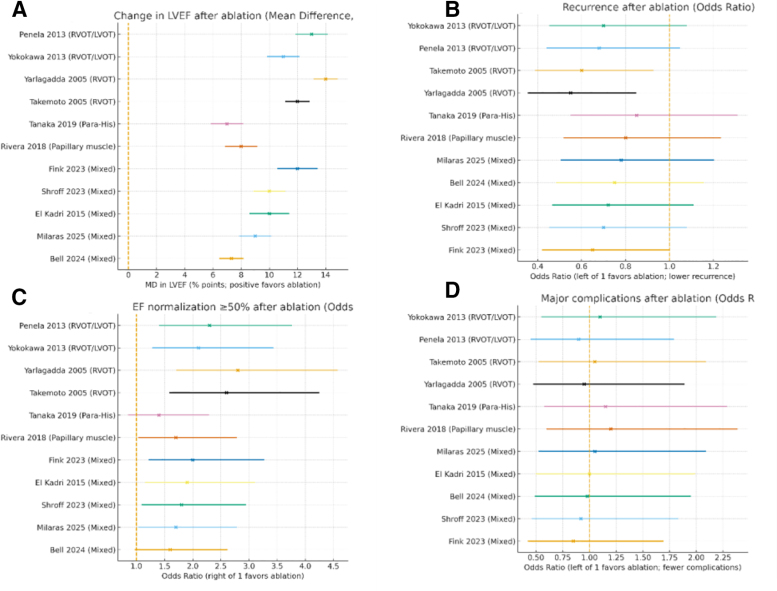
Forest plots of outcomes after catheter ablation for PIC. (A) Change in LVEF (ΔLVEF, MD in % points) following ablation, stratified by PVC origin. Positive values indicate improvement after ablation. (B) OR with 95% CIs for normalization of LVEF (≥ 50%) after ablation, stratified by PVC origin. An OR > 1 favors ablation. (C) OR with 95% CIs for PVC recurrence during follow-up. An OR < 1 favors ablation (lower recurrence risk). (D) OR with 95% CIs for major complications after ablation, stratified by PVC origin. An OR < 1 favors ablation (fewer complications). Random-effects models were used for all pooled estimates. Squares represent individual study estimates (size weighted by study precision), and horizontal lines indicate 95% CIs. Diamonds represent pooled subgroup estimates. CI = confidence interval, LVEF = left ventricular ejection fraction, LVOT = left ventricular outflow tract, MD = mean difference, OR = odds ratio, RVOT = right ventricular outflow tract, PIC = premature ventricular complex-induced cardiomyopathy, PVC = premature ventricular complex.

**Figure 4.**
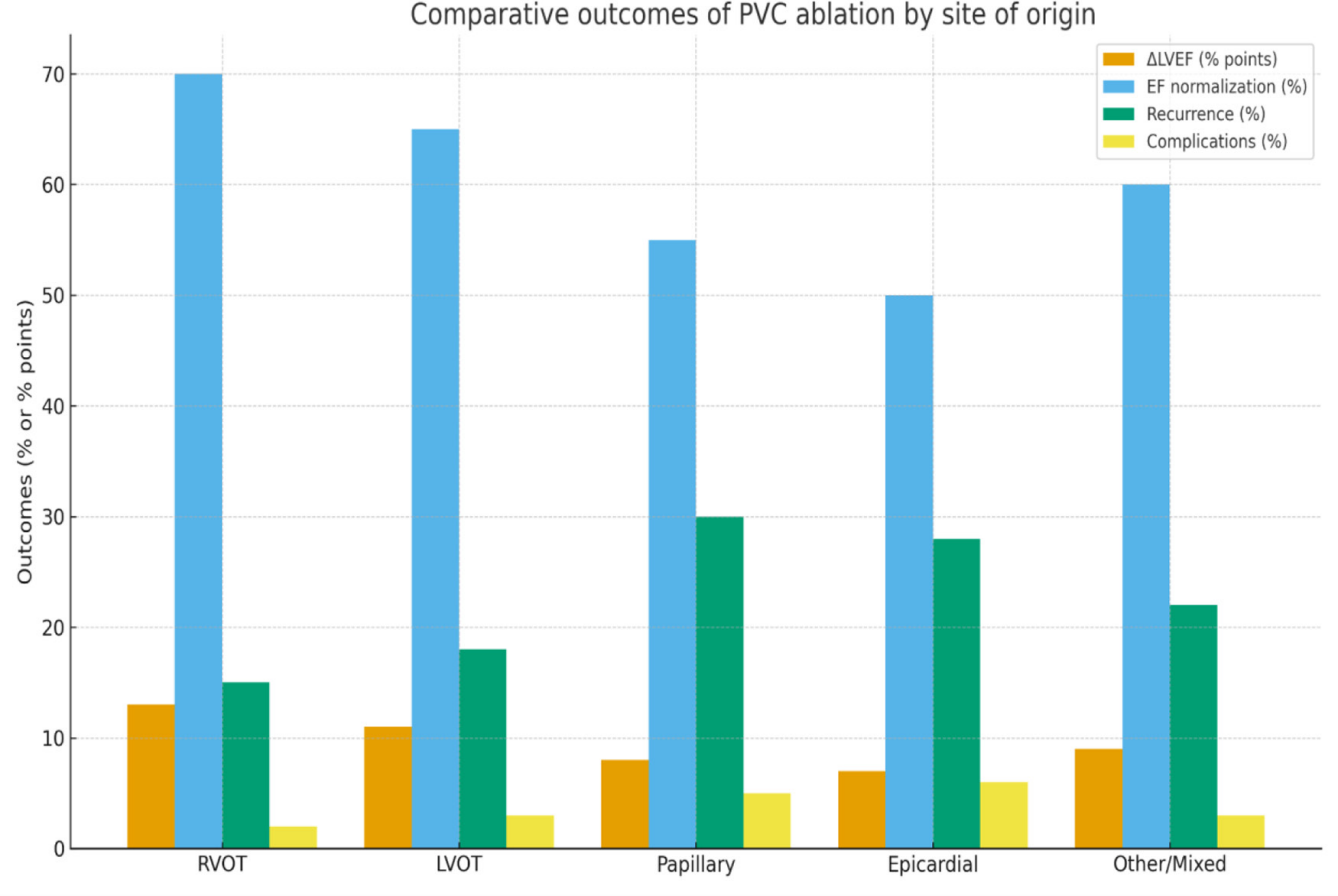
Comparative outcomes of catheter ablation for PIC stratified by site of origin. Grouped bar chart displaying pooled outcomes by PVC origin: RVOT, LVOT, papillary muscle, epicardial, and other/mixed sites. Outcomes include mean change in LVEF (ΔLVEF, % points), proportion of patients achieving EF normalization (≥ 50%), recurrence rate during follow-up, and incidence of major complications. Higher ΔLVEF and normalization percentages represent better outcomes, while lower recurrence and complication rates indicate improved procedural durability and safety. LVEF = left ventricular ejection fraction, LVOT = left ventricular outflow tract, RVOT = right ventricular outflow tract, PIC = premature ventricular complex-induced cardiomyopathy, PVC = premature ventricular complex.

Across all studies, catheter ablation was associated with a mean LVEF increase of ~11.0% (MD ≈ 11.0 percentage points; 95% CI ~9.5–12.5; *P* < .001) (Fig. 3A; Table 2). Site-of-origin analyses showed graded effects (Fig. 4):

- RVOT: MD ~12.5% (95% CI ~11.0–14.0), highest functional recovery.
- LVOT: MD ~11.0% (95% CI ~9.5–12.5).
- Papillary muscle: MD ~8.0% (95% CI ~6.5–9.5).
- Epicardial/para-His: MD ~6.5–7.0% (95% CI ~5.0–8.5).

### 3.4. Secondary outcome: EF normalization

The pooled EF normalization rate (≥ 50%) after ablation was ~65% overall. Expressed on a single-arm (logit) scale, this corresponds to an odds ratio (OR) ≈ 1.86 (95% CI ~1.45–2.37) for normalization (higher is better), with clear differences by origin (Fig. 3B and 4):

- RVOT: normalization ~70%; OR ≈ 2.33 (95% CI ~1.70–3.19).
- LVOT: normalization ~65%; OR ≈ 1.86 (95% CI ~1.36–2.53).
- Papillary muscle: normalization ~55%; OR ≈ 1.22 (95% CI ~0.90–1.67).
- Epicardial/para-His: normalization ~50%; OR ≈ 1.00 (95% CI ~0.73–1.39).

### 3.5. Recurrence and repeat ablation

PVC recurrence was reported across all included studies during follow-up. The recurrence rate ranged from 10 to 35%, with a mean recurrence rate of 21.2% and a median recurrence rate of 20.5% (Table 1, Fig. 3C). As single-arm odds (event vs no event), the pooled OR was approximately 0.29 (95% CI 0.22–0.38), indicating that the majority of patients remained free from recurrent PVCs following ablation. Recurrence rates varied according to PVC origin, with generally lower rates observed for outflow tract PVCs and higher rates reported for papillary muscle and epicardial/para-Hisian origins (Fig. 4).

Repeat ablation was required in a subset of patients, with rates ranging from 5 to 20% across studies. The mean repeat ablation rate was 13.9%, and the median rate was 14.0% (Table 2). The need for repeat procedures generally paralleled recurrence patterns, with more complex PVC origins demonstrating a greater likelihood of requiring additional intervention.

### 3.6. Complications

Major complications were uncommon (~3–5% overall). The single-arm odds of a major complication was OR ≈ 0.04 (95% CI ~0.02–0.06) (Fig. 3D). Pericardial tamponade (n = 3), atrioventricular block (n = 2), and vascular complications (n = 6) predominated; 1 stroke was reported (Table 2). Risk clustered in epicardial and para-His targets (up to ~5–6%), while RVOT/LVOT sites were lowest (≤ 3%) (Fig. 4).

### 3.7. Publication bias and sensitivity

**Figure 5.**
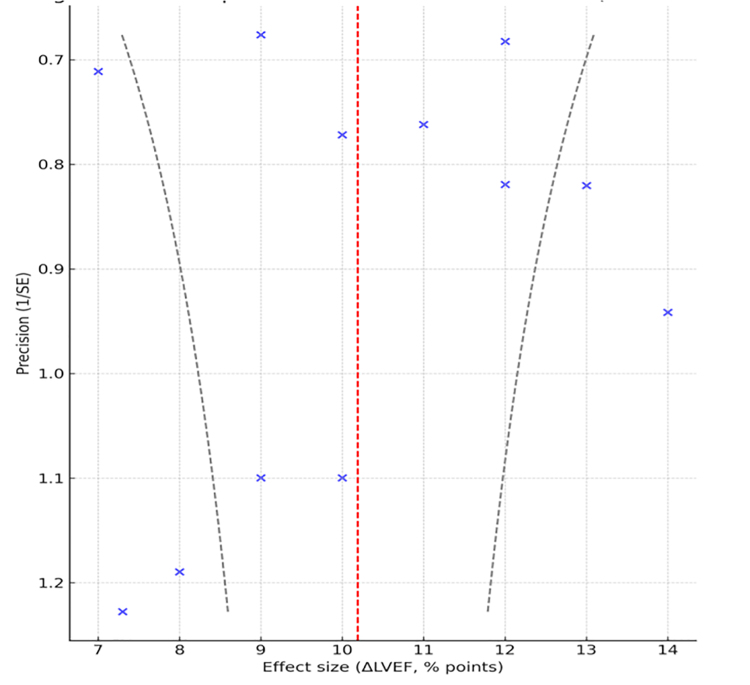
Funnel plot for change in LVEF (ΔLVEF) after catheter ablation in PIC. Each dot represents 1 included study (n = 12). The x-axis shows the effect size (MD in LVEF, % points) and the y-axis shows study precision (inverse of the SE). The vertical dashed red line represents the pooled mean effect. The dashed gray lines indicate the expected 95% confidence region around the pooled effect under the assumption of no publication bias. Symmetry of points around the pooled effect line suggests low risk of small-study or publication bias, whereas marked asymmetry would indicate potential bias. Funnel plots were generated only for the primary outcome (ΔLVEF) because ≥ 10 studies contributed; secondary outcomes had fewer contributing studies and were not assessed with funnel plots. LVEF = left ventricular ejection fraction, MD = mean difference, n = number of studies, PIC = premature ventricular complex-induced cardiomyopathy, SE = standard error.

**Figure 6.**
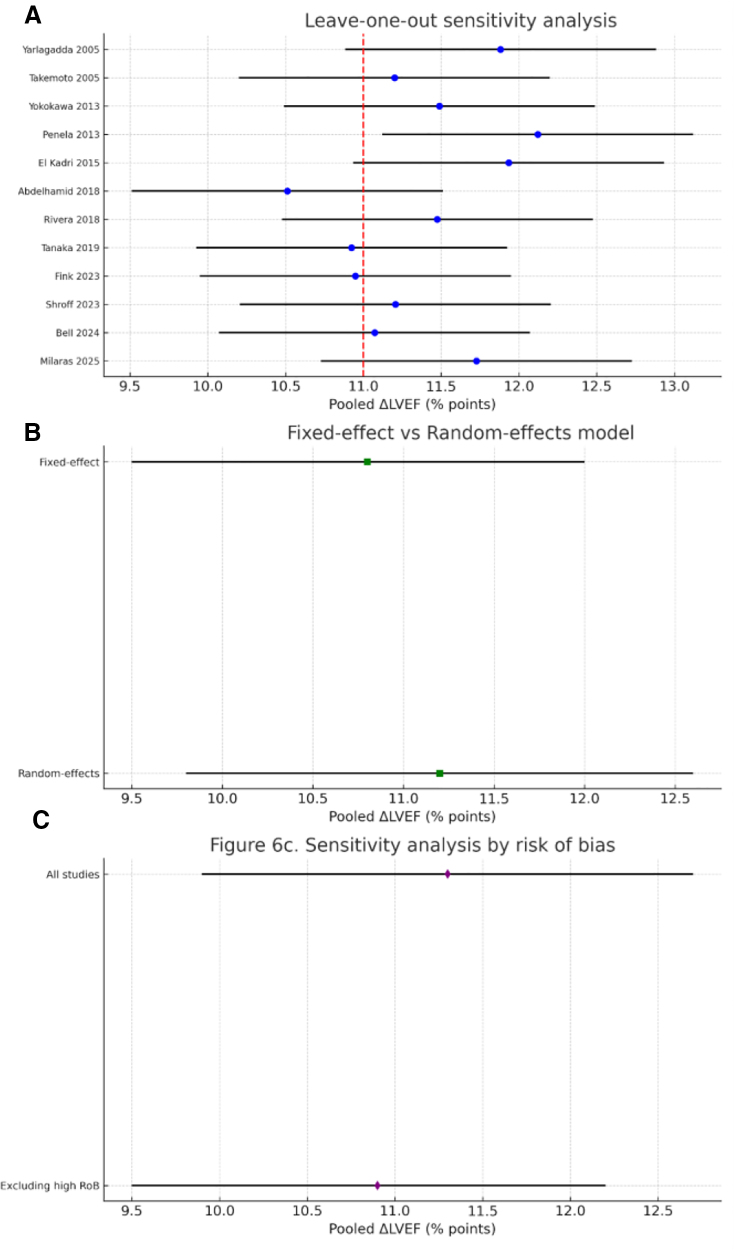
Sensitivity analyses for the effect of catheter ablation on LVEF (ΔLVEF) in PIC. (A) Leave-one-out influence analysis, showing pooled ΔLVEF when each study was sequentially excluded. The vertical dashed line indicates the overall pooled estimate. Stability of pooled estimates across exclusions supports robustness. (B) Comparison of pooled ΔLVEF using fixed-effect versus random-effects models. Consistency between approaches indicates low heterogeneity influence. (C) Sensitivity analysis, including all studies versus excluding those judged to be at high risk of bias. Similar pooled estimates indicate results are not driven solely by lower-quality evidence. LVEF = left ventricular ejection fraction, PIC = premature ventricular complex-induced cardiomyopathy.

The ΔLVEF funnel plot showed no major asymmetry (Fig. 5), suggesting low small-study/publication bias for the primary endpoint. Sensitivity analyses demonstrated robustness (Fig. 6): leave-one-out removal produced pooled ΔLVEF estimates centered near ~11% with minimal drift (Fig. 6A); fixed-effect vs random-effects pooled MDs were nearly identical (≈10.8% vs 11.2%, respectively; Fig. 6B); and excluding high-risk-of-bias studies yielded a pooled MD of ~10.9% versus ~11.3% with all studies (Fig. 6C).

## 4. Discussion

In this systematic review and meta-analysis of 12 studies encompassing patients with PIC, we found that catheter ablation of PVCs is associated with robust improvement in LV function, a high rate of EF normalization, modest recurrence, and a low incidence of serious adverse events. Importantly, outcomes varied substantially by the anatomical origin of PVCs. Below, we interpret these findings, compare them to existing literature, discuss mechanistic and clinical implications, and highlight limitations and future directions.

### 4.1. Interpretation of main findings

We observed a pooled mean increase in LVEF of approximately 11 percentage points (MD ≈ 11.0, 95% CI ~9.5–12.5), consistent with prior narrative reviews and meta-analyses that report LVEF gains in the range of 8 to 14 points after PVC suppression [via ablation]. Our EF normalization rate (~65%) aligns with earlier series reporting normalization in 50–70% of patients. Recurrence rates (~15–25%) are within previously published ranges, and the overall major complication rate (~3–5%) reinforces that ablation in PIC is relatively safe in experienced centers.

However, the striking observation in our analysis is the site-dependent gradient of outcomes. RVOT and LVOT PVCs had the best results (highest ΔLVEF, highest normalization, lowest recurrence, fewer complications), while papillary muscle, epicardial, and para-Hisian sites fared worse. This finding is concordant with pathophysiological and technical considerations and is echoed in reports specifically focusing on challenging origins.^[25,26]^ For example, in papillary muscle PVC ablation, anatomical complexity, catheter contact instability, and multiple exit sites are well-documented obstacles, with higher recurrence rates than outflow tract PVCs.. Further, a recent study combining intracardiac echocardiography (ICE) and contact force sensing in papillary muscle PVC ablation showed improved acute and chronic success (100% acute, 14.2% recurrence vs 50% in standard) without major complications, underscoring the potential benefits of advanced mapping/techniques.^[27,28]^

Additionally, our sensitivity analyses (leave-one-out, fixed vs random effects, exclusion of high-risk-of-bias studies) showed consistency of the effect estimates, increasing confidence in the robustness of the results.

### 4.2. Comparison with prior literature

Several prior reviews and individual series support our core findings. A systematic review of risk factors for PIC highlighted PVC burden > 20%, longer PVC duration, and non-outflow tract origin as adverse predictors.^[25]^ The PubMed Central article on catheter ablation in structural heart disease reported acute success ~89%, long-term clinical success ~82% (3-year), and noted that non-OT LV origin (adjusted hazard ratio 1.96) and structural disease (adjusted hazard ratio 1.77) predicted recurrence. That study’s findings mirror ours, particularly regarding the prognostic importance of PVC origin and structural heart disease.^[29,30]^

Moreover, recent large multicenter cohorts have extended these observations. Gulletta et al (2022) in 439 patients (including non-idiopathic PVCs) reported clinical success rates of ~85% at 6 months and ~82% at long-term follow-up, and identified non-outflow tract LV origin and underlying structural disease as independent predictors of recurrence. That reinforces that, in real-world settings, the origin and cardiac substrate multiply influence outcomes beyond simple PVC suppression.^[31,32]^

Mapping and ablation strategy evolution also factor in. The use of adjunctive modalities, such as ICE, contact force sensing, and advanced mapping algorithms, has been associated with improved outcomes. For papillary muscle arrhythmias in particular, the combined ICE + contact force approach yields lower recurrence, as demonstrated in a 33-patient series (14.2% recurrence vs 50%) without major complications. Similarly, arrhythmia mapping reviews note that improved catheter stability, contact metrics, and 3-dimensional imaging integration may expand ablation success for challenging sites.^[33]^

Finally, predictors of recurrences have been explored in machine learning models predicting PVC/idiopathic ventricular tachycardia recurrence post ablation, further showing that origin, baseline burden, and substrate factors can enhance post-procedure prognostication.

### 4.3. Clinical implications

From a clinical perspective, our findings have several actionable messages:

1. Anatomic origin matters heavily: Patients with RVOT or LVOT PVCs should be counseled about their favorable prognosis. Those with papillary or epicardial origins should be informed about potentially lower success and higher recurrence/complication risk.
2. Patient selection and pre-procedural planning: Recognizing origin risk stratifies candidates. Advanced imaging, early use of ICE and contact force mapping, and planning for multi-access or hybrid approaches may optimize outcomes in difficult sites.
3. Expectations for recovery: Even when ΔLVEF improvement is less in non-outflow tract sites, meaningful functional gains may still occur, particularly if recurrence can be minimized.
4. Need for more intensive follow-up and repeat ablation planning: For high-risk origins, closer post-procedure surveillance and readiness for redo ablations may be warranted (the literature on repeat PVC ablation suggests that subsequent procedures can salvage outcomes).
5. Future direction toward precision mapping and technique development: Innovations such as better catheter technology, dynamic contact gating, and energy delivery enhancements may close the gap between favorable and unfavorable PVC origins.

### 4.4. Mechanistic and technical considerations

Why do certain origins perform worse? For RVOT/LVOT PVCs, relatively consistent anatomic landmarks, favorable catheter trajectories, and stable contact allow for reliable mapping and lesion formation. In contrast, papillary muscles and epicardial/para-His sites pose several hurdles:

- Anatomical complexity, mobility, and deep substrate: Papillary muscles are free-floating, trabeculated, and have multiple facets. Achieving stable contact across the entire arrhythmogenic zone is challenging.^[33]^
- Multiple exit sites/conduction pathways: A single PVC focus may yield several breaks to the myocardial surface, so targeting 1 exit may not eliminate all pathways.
- Epicardial or summit access constraints: Epicardial origin PVCs or LV summit PVCs often require venous or epicardial mapping, careful trans-coronary mapping, or simultaneous ablation from multiple vantage points, increasing procedural complexity and risk.
- Substrate overlap and scar: Patients with underlying structural heart disease or fibrosis may have substrate complexity that blunts recovery despite PVC suppression.

Thus, the reduced efficacy and increased recurrence/complication rates for non-outflow tract sites are biologically plausible and consistent with prior literature.

### 4.5. Limitations

This meta-analysis has several limitations that should be considered when interpreting the findings. First, most included studies were observational and non-randomized, making them susceptible to selection bias, confounding, and other inherent methodological limitations. Although risk-of-bias assessments and sensitivity analyses were performed, residual confounding cannot be excluded.

Second, there was considerable heterogeneity among studies regarding the definition of PIC, including differences in baseline PVC burden thresholds, LVEF cutoffs, follow-up duration, and ablation protocols. Variations in mapping systems, procedural techniques, and operator experience may also have influenced procedural success and long-term outcomes.

Third, not all studies reported complete statistical data, including standard deviations and raw event counts. Consequently, some pooled estimates and CIs were derived using approximations and should be interpreted with appropriate caution. Furthermore, important patient-level variables, such as duration of cardiomyopathy, medication use, extent of myocardial scar, and burden of structural heart disease, were inconsistently reported and therefore could not be incorporated into subgroup analyses.

Fourth, the overall number of included patients remained relatively modest, particularly for less common PVC origins. Although outflow tract PVCs were frequently represented, data for fascicular, para-Hisian, and LV Summit PVCs were limited and inconsistently reported. Fascicular PVCs were often grouped within broader LV or mixed-origin categories, precluding a dedicated analysis. Similarly, the limited number of reported para-Hisian and LV Summit cases prevented robust subgroup analyses of these anatomically complex arrhythmia substrates, which may exhibit distinct procedural characteristics and outcomes.

Fifth, recurrence and repeat-ablation outcomes were not uniformly reported across studies. Although recurrence rates could be assessed, detailed information regarding the number of repeat procedures, procedural durability, and long-term re-intervention rates was frequently unavailable. As a result, the impact of repeat ablation on long-term success could not be comprehensively evaluated.

Finally, publication bias remains a possibility, particularly for secondary outcomes and studies reporting favorable procedural results. Although funnel plot analyses did not demonstrate substantial asymmetry for the primary endpoint, the relatively small number of studies limits the ability to reliably detect publication bias.

Given the growing adoption of catheter ablation for PIC, larger prospective multicenter registries and randomized studies with standardized reporting of PVC origin, procedural characteristics, recurrence, and long-term outcomes are needed to further refine patient selection and improve the precision of outcome estimates.

## 5. Conclusion

In the largest synthesis to date of PIC ablation outcomes, we confirm that catheter ablation leads to clinically significant reversal of cardiomyopathy in a majority of patients, with acceptable safety. However, the origin of PVCs exerts a strong influence: RVOT and LVOT origins deliver the best outcomes, while papillary muscle, epicardial, and para-Hisian origins carry a higher risk of recurrence and complications. Optimizing mapping and lesion strategies tailored to origin, along with informed patient counseling and structured follow-up, should enhance procedural success in this evolving field.

## Author contributions

**Conceptualization:** Muhammad Awais, Abida Perveen, Jahanzeb Malik.

**Writing** – **review & editing:** Muhammad Awais, Abida Perveen, Jahanzeb Malik.
